# A novel triage approach of child preventive health assessment: an observational study of routine registry-data

**DOI:** 10.1186/s12913-014-0498-0

**Published:** 2014-10-24

**Authors:** Janine Bezem, Meinou Theunissen, Simone E Buitendijk, Paul L Kocken

**Affiliations:** Department Preventive Youth Health Care, Municipal Health Service Gelderland-Midden, 6802 EJ Arnhem, The Netherlands; Department of Child Health, TNO, Leiden, The Netherlands; Leiden University, Leiden, The Netherlands; Department of Public Health and Primary Care, Leiden University Medical Centre (LUMC), Leiden, The Netherlands

**Keywords:** Triage, Task shifting, Efficient organization, Health service supply and distribution, Preventive youth health care, Preventive health assessments, Children

## Abstract

**Background:**

The coverage of preventive health assessments for children is pivotal to the system of preventive health screening. A novel method of triage was introduced in the Preventive Youth Health Care (PYHC) system in the Netherlands with an associated shift of tasks of professionals. Doctor’s assistants carried out pre-assessments to identify children in need of follow-up assessment, whereas in the traditional approach all children would have been screened by a doctor or nurse. The accessibility and care delivery of this new PYHC system was studied.

**Methods:**

The new triage approach was compared to the traditional approach in 780 children undergoing PYHC assessment with the use of an observational retrospective study design. Outcomes were attendance of assessment appointments (accessibility of care) and referral of children to either extra PYHC assessment or external specialised care (delivery of preventive care). PYHC registry data were analysed. In two regions of the Netherlands, 390 children five to six years of age were randomly selected from the PYHC registries according to the socio-economic strata of the schools they attended.

**Results:**

When the triage and traditional approaches to PYHC were compared, we found similar attendance rates for assessment appointments, namely about 90%. As expected, 100% of the children in the traditional group were assessed by a PYHC doctor compared to 46% of the children in the triage group. Significantly fewer children were referred for extra PYHC assessment or for treatment by an external specialised care giver when a triage as opposed to the traditional assessment approach was used (19.6% vs. 45.9%).

**Conclusions:**

The novel triage approach for preventive health assessment shows equal accessibility, but a different delivery of preventive care. A beneficial effect of the adoption of the triage approach is the opportunity to provide more attention from doctors and nurses to children at risk of health problems. However, lower referral rates of the triage approach may be explained by an under-identification of children with health problems. Further research is needed to document the health outcomes and the possible reduction of health care costs with a triage approach compared to traditional PYHC care.

**Electronic supplementary material:**

The online version of this article (doi:10.1186/s12913-014-0498-0) contains supplementary material, which is available to authorized users.

## Background

A preventive health care programme for children and young people can be found in most countries, with major attention being paid to immunisation, systematic screening for asymptomatic children and the detection of disorders [1,2]. Structural reforms of the health care systems in many countries today and increased attention to such health problems as mental health disorders and lifestyle issues are calling for changes to the system of preventive health care for children as well [3,4]. Preventive Youth Health Care (PYHC) services must be better aligned with current health priorities but must also address uneven access to care, inadequate programme quality and workforce shortages [5].

To meet these current health care needs of society, a novel approach has recently been developed for the provision of PYHC for children four to eighteen years in the Netherlands. This approach is based on triage and a shifting of the tasks among health care professionals. Triage can be defined as the process of determining clinical need, the likely response to intervention and the degree of urgency for such intervention [6]. The shifting of tasks can be defined as the delegation of existing tasks to current or new professionals who have less and/or more specific (i.e. tailored) training. Although triage and a shifting of the tasks of health care professionals have so far been introduced primarily in primary health care and emergency health care services, the integration of these principles in the PYHC system may have several promising advantages. These are: optimal use of the skills and expertise of health care professionals; reduced workloads of doctors and nurses; improved accessibility of health care and greater patient satisfaction [7-9]. To meet these current health care needs of society, a novel approach has recently been developed for the provision of PYHC for children in the Netherlands. This approach is based on triage and a shifting of the tasks among health care professionals.

The PYHC system of the Netherlands is unique. It has been offering routine preventive public health care to all Dutch children from birth to eighteen years of age for more than a hundred years. Access is free of charge and thus independent of insurance status. The Dutch PYHC has been aimed at monitoring the growth and development of children and at prevention of children's health problems. The system has been set up for preventive and screening services for asymptomatic children, including the provision of the national vaccination programme. A standard call-up scheme is utilised for this purpose. Data of children to be invited for an assessment are provided by municipal registries (zero to three years) or by schools (four to eighteen years). Traditionally, all children receive about seventeen routine health assessments, thirteen in the period from birth to three years (i.e. well baby clinics) and three times for the age group four to eighteen years (i.e. school health services). These assessments consist of a general physical examination including standardised screening procedures with regard to specific health related topics, and an interview with parents or with older children themselves concerning the child’s physical, developmental and psychosocial health. When problems are detected, PYHC doctors and nurses decide whether there is any need for advice, extra assessments by PYHC, or referral to specialised care. The specially trained community health care doctors, nurses and doctor’s assistants (henceforth: PYHC professionals) work separately from specialised clinical caregivers such as paediatricians or other clinical health professionals. PYHC professionals keep records on the routine health assessments in a registry system. The attendance rates for routine assessment are typically very high (i.e., more than 85% on average) [10,11]. The majority of children who are seen for such PYHC assessment show no health problems at the time. This raises the question of what frequency of routine PYHC assessment is most suitable and whether this must always be conducted by a doctor or a nurse. Some PYHC organisations in the Netherlands have introduced a triage approach to make the procedure for detecting children with health problems or at risk for health problems more efficient.

A two-step procedure has been adopted for children four to eighteen years of age in the Netherlands. In contrast to traditional PYHC, not all children are assessed by a doctor or a nurse in this new triage approach. Rather, children are seen by a doctor’s assistant who follows a strict pre-assessment protocol and refers only children with suspected health care needs for follow-up assessment by a PYHC doctor or nurse. Both pre-assessment and follow-up assessment are part of the triage health assessment procedure. This possibly creates time for PYHC doctors and nurses to devote their attention to children who need extra care, such as children with mental health and lifestyle related problems. More time in that case will be available for assessment of children on request of parents, teachers, professionals and children themselves.

**Figure 1:**
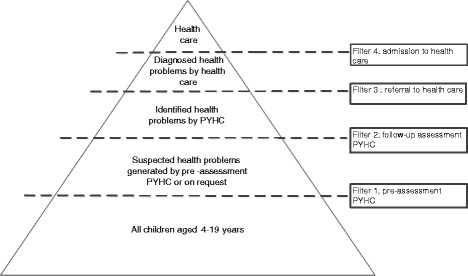
**Help-seeking process within triage approach to PYHC (adapted from Goldberg and Huxley, 1980, 1992).**

For a health screening programme it is essential that it is accessible for the population of children. Further, it should been assured that children are referred to the appropriate services according to their needs [12]. In this article, we report the results of a pilot study of the accessibility of PYHC assessment and delivery of preventive care by organisations that adopted the newly developed triage approach for PYHC in the Netherlands. As can be seen from Figure 1, the triage approach with PYHC pre-assessment by a specially trained assistant introduces an earlier filter to more intensive levels of health care [13,14]. This can possibly affect the access to PYHC assessment services by the public and delivery of PYHC care [15-19]. We therefore addressed the following research questions. What are the attendance (i.e. utilisation) rates for routine PYHC assessment when a triage as opposed to traditional approach is used? What are the rates of referral (i.e. delivery of care) when a triage as opposed to traditional approach is used?

The present pilot study was conducted to provide preliminary answers to these questions and is preparatory for future inquiries into the equity of service delivery and consequences for health outcomes of a triage approach to PYHC. We hypothesised the following: 1) With regard to attendance rates for routine PYHC assessment appointments, the triage and traditional approaches could be expected to produce equal results. This would indicate equal access to care. 2) With regard to the delivery of preventive care we hypothesised that triage may lead to fewer routine PYHC assessments by doctors as opposed to the traditional approach, as well as fewer indications for extra PYHC assessments and referrals to external specialised care givers.

## Methods

### Study sample

Attendance to PYHC assessment appointments and delivery of preventive care for two populations of children from separate geographic areas of the Netherlands were analysed. In a retrospective research design (see STROBE checklist, Additional file 1), we compared data from a total of 780 children. Random samples of 390 children aged five to six years were selected from the registries of two PYHC services in two geographically distinct regions, one using a triage approach and one using a traditional approach. Routine health assessments are being conducted by PYHC organisations in Dutch primary schools at two age groups namely five to six years and ten to eleven years which made access to registry data of a large number of children possible. We focused in this pilot study on the youngest age group of five to six years, for whom the detection of developmental problems is essential.

We selected a random sample of five to six year olds from the population of children who were invited for a pre-assessment (triage PYHC) or assessment (traditional PYHC). For each PYHC service, 390 children were selected from socio-economic strata of the schools being attended: 130 children from low SES schools, 130 from middle SES schools and 130 from high SES schools. The selection took place in a random way: the registers of the sample were ordered by day of birth and SES of school. Next, the first child out of five was selected. The socio-economic status of the schools was determined on the basis of national census statistics. Similar age and gender distributions were obtained for the triage PYHC assessment group (390 children from 78 schools) as for the traditional PYHC assessment group (390 children from 30 schools). The study sample was drawn from children undergoing assessment during a four month period in 2008.

### Triage approach versus traditional approach

**Figure 2:**
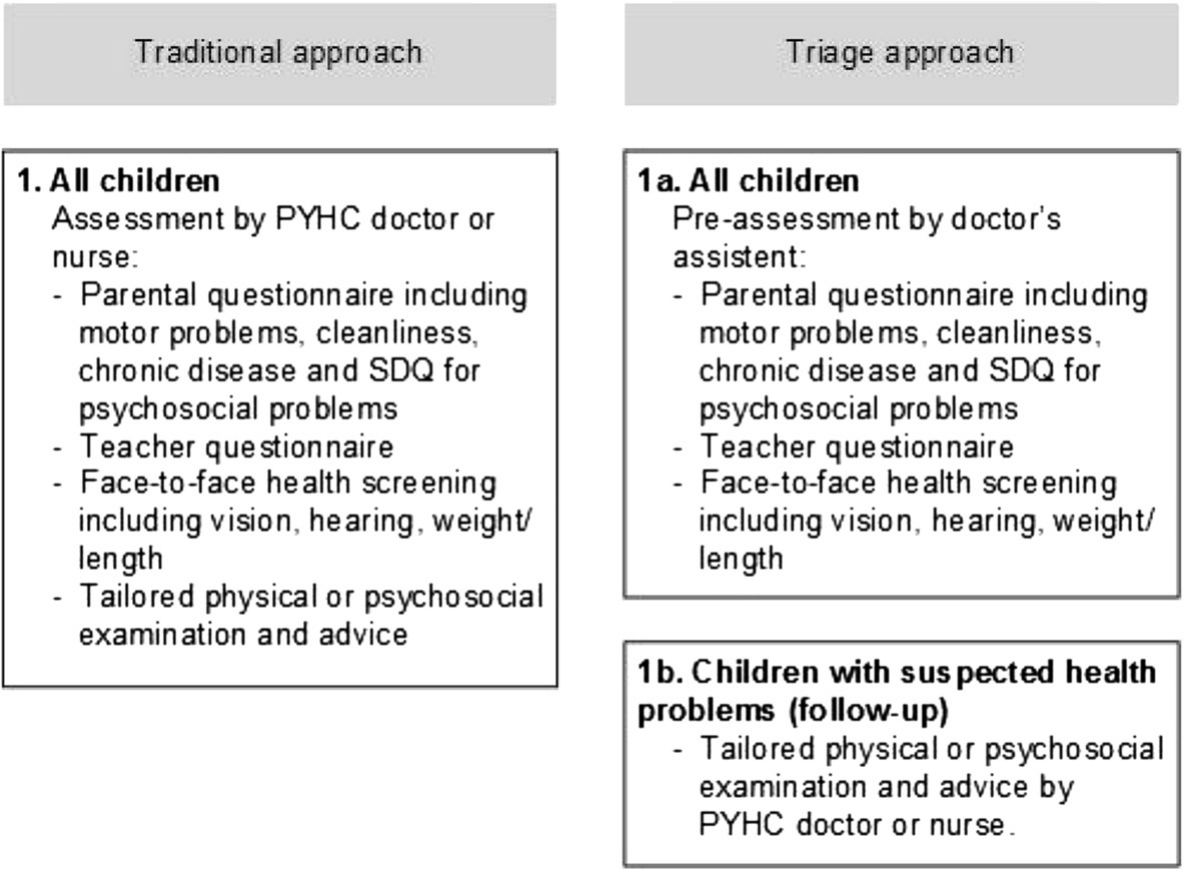
**Routine assessments: traditional approach versus triage approach.**

Pre-assessment of the children in the triage PYHC service was carried out by doctor’s assistants on the basis of the following information: PYHC records; questionnaires completed by school teachers and parents; and face-to-face screening. Routine assessments of the traditional approach versus the triage approach differ in certain aspects (see Figure 2).

The questionnaires covered a wide range of topics such as motor problems, cleanliness and chronic disease. The questionnaires included the Strength and Difficulties Questionnaire (SDQ) for parents in order to screen for psychosocial problems on the part of the child [20]. The assistants followed strict protocols to determine if follow-up PYHC assessment by the doctor or nurse was necessary. The nature and complexity of the suspected health problems determined whether follow-up assessment by a doctor or a nurse was needed: doctors attended to medical and developmental disorders; mostly nurses attended to psychosocial problems and lifestyle issues. During follow-up assessment by the PYHC doctor or nurse, the need for extra PYHC assessment or referral to an external service - a specialised care giver e.g., family doctor or social worker - was determined. The task of referral of children is assumed to be a vital part of the care delivered by PYHC.

Pre-assessment by the assistants was conducted in the schools in the absence of parents but with parental consent. Follow-up assessment by the doctor or nurse occurred in the presence of the child’s parent.

The children assessed by the traditional PYHC services were all examined by the PYHC doctor in the presence of the child’s parent. The doctors in the traditional group also had the following at their disposal: PYHC records and questionnaires completed by the teachers and parents prior to the consultation (see Figure 2). Those children with suspected problems were referred for extra assessment, which — just as in the triage approach — could be provided by the PYHC doctors or nurses themselves, or to an external service.

### Data collection

Data on PYHC assessment appointment attendance rates (i.e. accessibility of PYHC services) and the referral rates for extra PYHC assessment or to external specialised care givers (i.e. delivery of preventive care) were collected from the PYHC records. The extra PYHC assessment or external specialised care are called hereafter ‘extra care’.

Referral rates were determined for the following health indicators: psychosocial problems, visual disorders and overweightness. These health indicators were chosen because standard rules for screening for these health issues were available for both triage and traditional approaches to PYHC assessment. The psychosocial problems included behavioural and emotional problems on the part of the child, social interaction problems and/or child abuse. The identification of such psychosocial problems was based on the assessment made by the PYHC professional and the child’s SDQ scores [21]. Visual disorders, including amblyopia and impaired vision, were determined using a visual acuity test (i.e. the Snellen chart with SD scores based on the Dutch general population) [22]. Problems of overweightness were determined using the Body Mass Index. The child’s Body Mass Index (BMI) was derived from the PYHC records of routine health assessments. The thresholds used by the international obesity task force were adopted as the BMI cut-off points for overweightness and obesity [23]. SD scores for BMI were based on the Dutch general population [24].

Four of the 780 children had to be excluded because their data were incomplete. This left the data for a sample of 776 children to be analysed (390 traditional approach and 386 triage approach).

### Statistical analyses

First, we assessed differences in background characteristics between the two approaches using the Chi-square test and t-test. Next we compared the percentages of the children showing up for the assessment sessions in the traditional condition (usually assessment by a doctor) and the triage condition (pre-assessment by a doctor’s assistant and possibly follow-up assessment by a PYHC doctor or nurse) using the Chi-square test. We also compared the percentages of children referred for extra care for the two conditions. Referral rates for care were calculated for total problems, psychosocial problems, visual disorders and overweightness. We tested differences in referral rates for total problems for the two groups using four separate logistic regression analyses with referral to extra care (total, psychosocial problem, visual disorder, overweightness) as the outcome variables and the group and significant background characteristics (Table 1) as the independent variables (SPSS 22.0 for Windows, SPSS Inc., Chicago, IL).

**Table 1:** **Characteristics of children assessed by traditional versus triage approach**

		**Traditional approach**	**Triage approach**
		**(N = 390)**	**(N = 386)**
Age (years)*, M (SD)		5.7 (0.8)	6.3 (0.3)
Gender, N (%)	Boy	205 (52.6)	199 (51.6)
	Girl	185 (47.4)	187 (48.4)
Socio-economic status (SES), N (%)	Low	130 (33.3)	129 (33.4)
	Middle	130 (33.3)	130 (33.7)
	High	130 (33.3)	127 (32.9)

*Statistically significant at p <0.01 (t-test).

### Ethics

This study was approved by the internal TNO Review Board and is in accordance with the Dutch act on Medical Research Involving Human Subjects. Medical ethical approval was not required for this study.

## Results

Table 1 presents the background characteristics of the children participating in the study. The study groups did not differ significantly in terms of gender and socio-economic status, but the mean age of the children differed between the two approaches, 5.7 years for the traditional approach and 6.3 years for the triage approach.

The appointment attendance of the traditional assessment has been compared with the appointment attendance of pre-assessment and follow-up assessment of the triage approach. Our results show no significant different appointment attendance rates for the two approaches to PYHC.

As can be seen from Table 2, 351 of the sample of 390 children (90.0%) who were invited for an assessment in the traditional group, actually attended this assessment. In the triage group 372 of the sample of 386 children (96.4%) attended a pre-assessment by a doctor’s assistant and 143 of the sample of 163 children (87.7%) who were referred to a follow-up assessment by the doctor or nurse indeed attended this assessment.

**Table 2:** **Rates of appointment attendance of traditional and triage approach to preventive youth health care (PYHC) assessment**

	**Traditional approach**	**Triage approach**	
	**Assessment by PYHC doctor**	**Pre-assessment by PYHC doctor’s assistant**	**Follow-up assessment by PYHC doctor or nurse**
Appointment for assessment, N	390	386^a^	163^b^
Appointment attendance, N (%)	351 (90.0)	372 (96.4)	143 (87.7)

^a^Four children were excluded from analyses due to incomplete data.

^b^Eight children did not receive a call for follow-up assessment while being positively assessed by the assistant.

All of the children in the traditional group received routine PYHC assessment by a doctor while only 46% of the children in the triage group required PYHC assessment by a doctor or a nurse. Next, the percentages of children referred for extra care were compared (see Table 3). A significant difference was found: 45.9% for the traditional group were referred to extra care as compared to 19.6% for the triage group (OR = 3.9, 95% CI = 2.7-5.8).

**Table 3:** **Rates of follow-up assessment and referral for extra care of the traditional and triage approach**

	**Traditional approach (N = 351)**	**Triage approach (N = 372)**			
	**Referral for extra care, after assessment by PYHC doctor**	**Follow-up assessment by PYHC doctor or nurse, after pre-assessment by PYHC doctor’s assistant**	**Referral for extra care, after follow-up assessment by PYHC doctor or nurse**		
	N (%)	N (%)	N (%)	OR	95% CI
Total*	161 (45.9)	171 (46.0)	73 (19.6)	3.9	2.7-5.8
Psychosocial problem	28 (8.0)	59 (15.9)	19 (5.1)	1.5	0.7-3.0
Visual disorder*	29 (8.3)	36 (9.7)	12 (3.2)	3.0	1.5-6.1
Overweightness*	43 (12.3)	30 (8.1)	20 (5.4)	3.6	1.9-6.7

*Statistically significant at p <0.01 (logistic regression analyses).

The percentages of children referred to extra care also differed significantly for the health indicators visual disorder and overweightness between the traditional versus triage group. For possible visual impairments, 8.3% of the children in the traditional group were referred to extra care, compared to 3.2% of the children in the triage group (OR = 3.0, 95% CI = 1.5-6.1), after 9.7% had seen a PYHC doctor or nurse for follow-up assessment. For possible problems of overweightness, 12.3% of the children in the traditional group were referred to extra care, compared to 5.4% in the triage group (OR = 3.6, 95% CI = 1.9-6.7), after 8.1% had seen a PYHC doctor or nurse for follow-up assessment. No difference was found for the health indicator psychosocial problems. For suspected psychosocial problems, 8.0% of the children in the traditional group were referred for extra PYHC or external care compared to 5.1% of the children in the triage group (OR = 1.1, 95% CI = 0.7-3.0), after 15.9% in this group had seen a PYHC doctor or nurse for follow-up assessment.

The two approaches also differed in terms of the proportions of children who were referred to extra care by PYHC and those who were referred to specialised care outside of PYHC. In the traditional approach 39.9% of 351 children were referred to extra care by PYHC, versus 14.8% of 372 children who received triage approach (OR = 4.5, 95% CI = 3.0-6.7). 12.5% of 351 children in the traditional approach versus 5.1% of 372 children in the triage approach were referred to specialised care (OR = 2.4, 95% CI = 1.3-4.7).

## Discussion

In this study, a novel method of triage for the public health assessment of children combined with a shifting of the tasks of Preventive Youth Health Care (PYHC) professionals was explored. We compared the attendance rates for the PYHC assessment appointments in groups using a triage approach versus a traditional approach. PYHC appointment attendance rates were taken to be indicators of the accessibility of PYHC. We also examined the referral rates for extra PYHC assessment or external specialised care, called ‘extra care’, as indicators of delivery of preventive care, assuming that referral is a vital part of the care delivered by PYHC.

The type of approach, i.e. a triage or traditional approach did not affect the accessibility of the routine PYHC assessment. The appointment attendance rates for PYHC assessment, which are traditionally quite high, continued to be high also for the triage approach to assessment. The attendance rate of the pre-assessment appointments were probably high because the parents were not required to be present.

Major differences in the referral rates for extra care were detected when the traditional approach was compared to the triage approach: lower referral rates for extra care were found for the triage approach relative to the traditional approach. The different referral rates for extra care can most likely be attributed to the different processes used to identify health problems in the two approaches. In the traditional assessment approach, all children are assessed by a PYHC doctor or nurse. In the two-step, triage approach to assessment, all children are pre-assessed by a doctor’s assistant and only those in need of follow-up (i.e. with health problems or at risk for health problems) are referred for assessment by a PYHC doctor or nurse. It is possible that this two-step approach provides an additional barrier to access to care. Children with health problems may be under-identified (false negatives, i.e. incorrectly classified as healthy) and therefore not referred for extra care. Another explanation for the lower referral rates is that in the second step in the triage assessment process, the PYHC doctor or nurse can provide more tailored advice, recommendations and reassurance, which can remove the need for further referral to extra care. It is, of course, also possible that spontaneous remission occurs during the period between pre-assessment by the doctor’s assistant and follow-up by the PYHC doctor or nurse and that this reduces referral for extra care in the triage group in particular. We did not measure the care which may have been sought during the period between pre-assessment and follow-up assessment, although this could also account for the significantly lower rate of referral for extra care in the triage group compared to the traditional group. Finally, the different referral rates found for extra care in the two groups might lie in earlier identification of health problems in the triage group as the triage approach to assessment allows for more responding to requests and questions from parents, teachers and the children themselves and may therefore nip more problems in the bud than a traditional approach to assessment.

When we compared the referral rates for extra care for psycho social problems, visual disorders and overweightness in our study to the actual prevalence rates for these problems among five and six year olds in the Netherlands, the triage referral rates resembled the actual prevalence rates of six percent for psychosocial problems and two to four percent for visual disorders [8,25]. The traditional-group referral rate of twelve percent for extra care for overweightness was higher than the triage group referral rate of five percent, but approached the actual prevalence rate of fifteen percent among five and six year olds in the Netherlands [26,27]. The referral rates for both the triage and the traditional groups in the present study represent health problems which have been newly identified by the PYHC service while the actual prevalence rates include problems which are already known. This means that PYHC referral rates for extra care may be lower than prevalence rates. More detailed and large-scale research on PYHC assessment practices and approaches is needed to gain insight in the identification of care needs and subsequent referrals.

### Strengths and weaknesses of the present study

A strength of the present study is that we were able to carefully compare the traditional and triage approaches by matching the groups with regard to the spread of socio-economic backgrounds (i.e. equal numbers of low, middle and high SES children in each group). We included a homogenous group of children within the age range of five to six years and controlled for differences in age distribution between the two study groups. Another strength is that we analysed assessment for a limited number of health problems for which standard screening guidelines have been established. Both of the approaches studied here thus used similar screening methods, which limited the possibility of observation bias.

A possible limitation of this study is the lack of insight into the numbers of children correctly and incorrectly identified with a problem in the triage versus traditional approaches to routine PYHC assessment. In this pilot work, we did not monitor the results of the referrals for extra care, and we therefore do not know if children were incorrectly referred for a health problem or potential health problem. For that matter, we do not know if children with actual health problems were mistakenly missed. Another possible limitation is the use of a retrospective research design. Marked differences in the identification and/or reporting of health problems by PYHC professionals cannot be ruled out and may have influenced our results. A last limitation is the inclusion of only two PYHC organisations in this study. A larger sample of organisations could add to the robustness of the data set and validity of the outcomes presented.

### Implications for preventive youth health care and directions for future research

This study provided a preliminary indication for the triage approach to have introduced a shift of tasks among PYHC professionals without sacrificing accessibility of PYHC assessment (i.e. attendance rates). The shifting of tasks with the introduction of pre-assessment by doctor’s assistants and fewer referrals to extra care of PYHC resulted in a less time consuming PYHC assessment procedure. This triage procedure enables PYHC doctors and nurses to devote more attention to children with special health care needs, often related to social inequities, mental health and lifestyle related problems. Time can be given for other consultations than the routine assessments, such as on request of parents, youths themselves or school staff. In this study we did not investigate the PYHC consultations at the request of schools, parents or children themselves.

The shift of tasks to PYHC doctor’s assistants within a triage approach to assessment, calls for new competencies on the part of these PYHC professionals and may result in the loss of generalised knowledge and expertise on the part of PYHC doctors and nurses when not all children are seen by them. Training of PYHC professionals is thus needed to maximise their diagnostic skills [28,29]. Considerable attention has been paid to the training of all PYHC professionals working with a triage approach to routine PYHC assessment, but research is needed to determine the actual quality of detection using such an approach. A criterion for determining the quality of detection could be the diagnosis of problems by professionals from an external organisation. This would allow us to determine the accuracy of referral for extra care (i.e. justified or not justified) and the quality of a triage approach to routine PYHC assessment in general. Examination of the outcomes of referrals for extra assessment by PYHC professionals or external specialised care givers can give us insight into the extent of compliance with such referral. It also can provide insight into the equity of care distribution to the children who are in need of health care.

Research across a greater age range and greater number of PYHC organisations using nevertheless uniform protocols and standard registration procedures to reduce the possibility of observation bias, is needed. Research is also needed to document the satisfaction of the children, young people, their parents and their teachers with a triage approach to routine PYHC assessment and the resulting care. Moreover, research into the effects of the new triage approach on the long-term need for care is advised. Finally, research into the costs of the new triage approach compared to routine PYHC assessment reported on here must be undertaken, particularly with respect to the traditional PYHC assessment approach.

## Conclusions

The present results show that a triage approach compared to routine PYHC assessment maintains the accessibility of assessments. The use of doctors and nurses for routine assessments has been reduced through a shift of assessment tasks among the PYHC professionals. The delivery of preventive care to children, including referral to external services has changed in the new approach. The triage approach for PYHC assessment may create opportunities for greater attention from doctors and nurses to children who are at risk and to children with clear health needs. The triage approach for routine PYHC assessment and its contribution to efforts in reducing the need for specialised health care among children and into adulthood needs further validation.

### Availability of data

Anonymised data can be provided by TNO to researchers on request.

## Additional file

Additional file 1:
**STROBE checklist for cohort studies (adapted from** http://www.strobe-statement.org/index.php?id=available-checklists).

## Abbreviations

BMI: Body Mass Index
SDQ: Strength and Difficulties Questionnaires
SES: Socio-economic status
PYHC: Preventive Youth Health Care
